# Gaps in Documenting Interpreter Service Utilization Among Emergency Department Clinicians Treating Patients With Limited English Proficiency

**DOI:** 10.1111/acem.70361

**Published:** 2026-06-12

**Authors:** Michael Gottlieb, Aamina Naveed, Emily Nguyen, Ildemaro J. González, Carlos Olvera, Giles W. Slocum

**Affiliations:** ^1^ Department of Emergency Medicine Rush University Medical Center Chicago Illinois USA; ^2^ Rush Medical College Chicago Illinois USA; ^3^ Rush University Medical Center Chicago Illinois USA

Language concordance between clinicians and patients is a critical component of safe and effective emergency care. Patients with Limited English Proficiency (LEP) are at increased risk for communication errors, misunderstanding of diagnoses and treatment plans, and lower satisfaction with care [1]. These challenges are particularly pronounced in the emergency department (ED), where time constraints, high acuity, and limited prior relationships heighten the importance of clear communication [2, 3, 4]. Data suggest ED patients with LEP have higher rates of admission and unplanned revisit within 72 h [5].

Federal law requires that health systems receiving federal funding provide meaningful access to language services for patients with LEP [6, 7]. Professional interpreter services, whether in‐person, telephonic, or video‐based, are widely recognized as the standard for facilitating communication in clinical encounters when language discordance exists. Despite these requirements, interpreter utilization remains inconsistent across health care settings [8]. Even when interpreters are used, documentation of their involvement can be variable. Accurate documentation serves multiple purposes, including demonstration of regulatory compliance, supporting continuity of care, facilitating quality monitoring, and may provide medicolegal protection.

The electronic health record (EHR) represents a primary mechanism for capturing interpreter use. Many institutions require clinicians to indicate whether an interpreter was used and to document identifying information, such as the interpreter's name or identification number. However, limited data exist regarding the reporting and completeness of interpreter documentation in ED settings, particularly within systems that mandate interpreter notation in physician documentation.

The objective of this study was to assess documentation of interpreter service utilization among patients with LEP presenting to the ED within a single healthcare system. We sought to quantify the frequency of documented interpreter use, determine the extent to which required elements were completed, and identify factors associated with reduced documentation.

We performed a retrospective chart review of ED encounters within a single integrated healthcare system over a 12‐month period from October 1, 2024, through September 30, 2025. The Rush University Medical Center ED is located in an urban setting within the Midwest and has an annual volume of approximately 68,000 ED patients. The ED includes a 3‐year emergency medicine residency program and is a level two trauma center and comprehensive stroke center. Institutional policy requires documentation of interpreter use within emergency physician notes and all EHR notes include a required interpreter field noting if an interpreter was utilized and their name or identification number. The institution uses Epic (Epic System Corporation; Verona, WI) as the EHR. At the time of this study, ED interpreter services were primarily provided via telephone or video‐based options. The study adheres to the STROBE guidelines [9], and was reviewed and approved by the Rush University institutional review board with a waiver of informed consent due to its retrospective nature and minimal risk to participants.

We performed a query of all ED encounters during the study period filtered by language. Patient primary language is a required entry within Epic for all patients entered at the time of registration. We included all adult (age ≥ 18 years) ED patient encounters for whom a primary language other than English was listed in Epic in the language field. Encounters were excluded if the patient was documented as bilingual with English listed among preferred languages, left without being seen or were transferred to another location (e.g., obstetrics unit) prior to ED evaluation, was unresponsive throughout the encounter, or lacked sufficient encounter details for review. Encounters with no language recorded were also excluded.

The research team developed and piloted a standardized data abstraction tool and codebook. Prior to full abstraction, both reviewers independently evaluated 20 randomly selected charts to assess clarity of definitions and consistency of interpretation. Interrater reliability was 100% for all abstracted variables in this pilot sample. After refinement of the abstraction tool, the remaining charts were divided and reviewed independently.

For each encounter, the following variables were collected: ED arrival date and time, age, sex, race, ethnicity, preferred language, documentation of interpreter use (defined as either the interpreter field listing that an interpreter was not used or the interpret field having been manually deleted by the note writer), and documentation of interpreter name or identification number. Data were obtained by reviewing both the patient demographic sheet and all emergency physician notes occurring during the duration of the encounter. When notes indicated that no interpreter was used, reviewers captured any documented reason, such as patient refusal, and whether the family interpreted for the patient.

Data were summarized using descriptive statistics and analyzed using multivariable logistic regression. The primary dependent variable was interpreter use (yes/no). Independent variables included age, sex, race, ethnicity, preferred language, arrival time category, weekend arrival, and ED disposition. Secondary descriptive outcomes included documentation of interpreter identification numbers, patient refusal of interpreter services, and use of family members as interpreters among encounters without documented interpreter use. Bivariate analyses were conducted using chi‐square tests and independent‐samples *t*‐tests. Collinearity was assessed using variance inflation factors. All prespecified variables were entered simultaneously into the final model. Adjusted odds ratios (aOR) with 95% confidence intervals (CIs) are reported. Analyses were performed using the Statistical Package for the Social Sciences version 29.0.2.0 (SPSS Inc., Armonk, NY).

There were 68,608 total ED encounters during the study period. Of those, a total of 8184 (11.9%) were among patients with a non‐English preferred language listed in Epic. Of these, 5665 (69.2%) encounters met inclusion criteria. Exclusions included 1241 encounters in which patients were bilingual with English listed among preferred languages, 964 encounters in which patients left without being seen or were transferred to another location prior to evaluation, 224 encounters lacking any recorded language, and 90 encounters involving unresponsive patients.

The mean age of included patients was 61.5 years, and 56.5% were female (Table 1). Spanish was the most common preferred language accounting for 78.4% of encounters, followed by Cantonese (9.2%), Mandarin (3.6%), and Polish (1.9%). Additional languages each comprised smaller proportions of the cohort (Appendix S1).

**TABLE 1:** Demographics of emergency department patients with limited English proficiency.

Demographic	*N* (%) or Mean ± SD
Age (years)	61.5 ± 18.8
Sex	
Female	3201 (56.5%)
Male	2464 (43.5%)
Race	
Asian	704 (12.4%)
Black or African American	65 (1.1%)
Other race	3554 (62.7%)
White	1259 (22.2%)
Not reported	83 (1.5%)
Ethnicity	
Hispanic or Latino	4324 (76.3%)
Not Hispanic or Latino	1327 (23.4%)
Not reported	14 (0.2%)
Preferred language	
Spanish	4440 (78.4%)
Not Spanish^a^	1225 (21.6%)
Day of week	
Weekday	4179 (73.8%)
Weekend	1486 (26.2%)
Arrival time	
0:00–05:59	432 (7.6%)
06:00–11:59	1675 (29.6%)
12:00–17:59	2217 (39.1%)
18:00–23:59	1341 (23.7%)
ED disposition	
Admitted	670 (11.8%)
Discharged	3087 (54.5%)
Expired	3 (0.1%)
Extended ED observation	1858 (32.8%)
Left against medical advice	33 (0.6%)
Transferred	14 (0.2%)

Abbreviations: ED, emergency department; SD, standard deviation.

^a^ The distribution of non‐Spanish languages is available in Appendix S1.

Of the 5665 encounters, 2787 (49.2%) documented any use of an interpreter in the EHR. Among those reporting use of an interpreter, 667 (23.9%) recorded the interpreter identification number or name. Among those who did not document the use of an interpreter (*n* = 2879), 512 (17.8%) reported that the patient declined interpreter services, while 596 (20.7%) reported using a family member. The remaining 1770 (61.5%) did not document interpreter use or a reason for not using an interpreter.

When analyzing those with versus without a documented interpreter use, there were statistically significant differences in age, race, ethnicity, preferred language, day of presentation (weekend versus weekday), and disposition on bivariate analysis (Appendix S2). There was evidence of collinearity between ethnicity and preferred language, so ethnicity was withheld from the multivariate analysis (Appendix S3). In multivariable logistic regression, there were lower odds of documented interpreter use with increasing age (aOR: 0.99 per year; 95% CI 0.99–0.99) and among White patients (aOR: 0.85; 95% CI 0.85–0.74–0.97), while there were higher odds of documented interpreter use among Asian patients (aOR: 1.66; 95% CI 1.30–2.13). Documented interpreter use was also less likely on weekends (aOR: 0.85; 95% CI 0.75–0.96) and among those who were admitted (aOR: 0.68; 95% CI 0.57–0.81) or placed in extended ED observation (aOR: 0.84; 95% CI 0.75–0.95; Appendix S4).

In this retrospective review of ED encounters involving patients with LEP, we found that documentation of interpreter service utilization was inconsistent and frequently incomplete. Approximately half of ED encounters lacked explicit documentation of any interpreter use, despite institutional requirements to record this information in physician notes. This is consistent with a prior study of 1266 patients across two Canadian EDs, which found that 52% reported interpreter utilization [10]. Among encounters in which interpreter use was documented, fewer than one‐quarter included the interpreter name or identification number.

These findings highlight a gap between institutional documentation requirements and recorded practice. While the absence of documentation does not necessarily indicate that interpreters were not used, failure to record interpreter involvement undermines the ability of health systems to demonstrate compliance with federal requirements and to evaluate the adequacy of language services. Without accurate documentation, quality improvement efforts aimed at enhancing language access cannot be effectively guided.

Incomplete documentation also has implications for continuity of care. ED encounters often serve as entry points into the healthcare system. Subsequent members of the healthcare team rely on prior documentation to understand communication barriers, patient preferences, and prior use of language services. When interpreter use is not recorded, future clinicians may be unaware of ongoing language needs or prior refusals, potentially leading to inconsistent provision of services.

Consistent with prior work [11], we also found that interpreter use was less commonly reported among older patients, with a progressive decline with each year of age. This may reflect disparities based on age, patient preferences, or more access to family performing interpretations for patients. We also identified lower rates on weekends, which may reflect differences in family member availability or ED volumes. Among those admitted or in extended ED observation, interpreter use was lower, which may reflect the perception of another clinician assuming care and transfer of responsibility versus a reduced perception of risk since the patient was not discharged.

The low rate of entry of interpreter names or identification numbers suggests that required documentation fields may not be consistently enforced or that workflow barriers impede completion. In the busy ED setting, clinicians may prioritize clinical decision‐making over documentation details that may be perceived as administrative. The EHR design may also contribute, particularly if required fields can be bypassed or auto‐populated without verification. Interventions such as hard stops, automated prompts linked to preferred language fields, or integration of interpreter service logs with clinical documentation may improve capture of interpreter utilization.

Our study has several important limitations. It was conducted within a single healthcare system and may not be generalizable to other institutions with different documentation workflows or interpreter service models. The retrospective design precludes assessment of actual interpreter use independent of documentation. We were unable to independently verify whether encounters without documentation of an interpreter involved ad hoc interpreters, bilingual staff, or informal family interpretation, which may also have implications for quality and compliance. Additionally, we did not specifically evaluate the extent of interpreter use. While we used a primary outcome of any interpreter use, it is unclear the degree to which interpreters were used throughout the clinical encounter among those clinicians documenting interpreter use in at least one clinical note. Moreover, we relied upon EHR documentation of language based upon their preferred language not being English but did not directly assess language proficiency of the ED patients. Finally, we did not assess clinical outcomes associated with documented or undocumented interpreter use.

Our findings identify incomplete documentation of interpreter utilization as a measurable systems issue in emergency care. Future studies should examine whether improvements in documentation correlate with consistent interpreter use and patient‐centered outcomes.

## Funding

The authors have nothing to report.

## Disclosure

The authors have nothing to report.

## Supporting information


**Appendix S1:** Distribution of languages among those with limited English proficiency.
**Appendix S2:** Bivariate analyses among those with versus without interpreter use.
**Appendix S3:** Assessment of collinearity.
**Appendix S4:** Multivariable logistic regression among those with versus without interpreter use.

## Data Availability

The data that support the findings of this study are available from the corresponding author upon reasonable request.
