# Supplementary material for: Gaps in Documenting Interpreter Service Utilization Among Emergency Department Clinicians Treating Patients With Limited English Proficiency
Source: Acad Emerg Med. 2026 Jun 12;33(6):e70361. doi: 10.1111/acem.70361 (PMC13263409; doi:10.1111/acem.70361)
Supplement: Supplementary file 1 — Appendix S1: Distribution of languages among those with limited English proficiency. Appendix S2: Bivariate analyses among those with versus without interpreter use. Appendix S3: Assessment of collinearity. Appendix S4: Multivariable logistic regression among those with versus without interpreter use. [file ACEM-33-0-s001.docx]

**APPENDIX**

**Appendix 1. Distribution of languages among those with limited English proficiency**

| **Language** | **n (%)** |
| --- | --- |
| Spanish | 4,440 (78.4%) |
| Cantonese | 523 (9.2%) |
| Mandarin | 204 (3.6%) |
| Polish | 106 (1.9%) |
| Sign Language | 49 (0.9%) |
| Arabic | 46 (0.8%) |
| Toishanese | 37 (0.7%) |
| Ukrainian | 37 (0.7%) |
| Russian | 31 (0.5%) |
| Greek | 19 (0.3%) |
| Romanian | 14 (0.2%) |
| Vietnamese | 13 (0.2%) |
| Urdu | 13 (0.2%) |
| French | 13 (0.2%) |
| Italian | 12 (0.2%) |
| Korean | 9 (0.2%) |
| Thai | 7 (0.1%) |
| Farsi | 7 (0.1%) |
| Albanian | 7 (0.1%) |
| Portuguese | 7 (0.1%) |
| Haitian Creole | 6 (0.1%) |
| Tactile Sign Language | 4 (0.1%) |
| French Creole | 4 (0.1%) |
| Telugu | 4 (0.1%) |
| German | 4 (0.1%) |
| Tigrinya | 4 (0.1%) |
| Pashto | 4 (0.1%) |
| Lithuanian | 3 (0.1%) |
| Uzbeck | 3 (0.1%) |
| Hindi | 3 (0.1%) |
| Filipino | 3 (0.1%) |
| Tagalog | 2 (<0.1%) |
| Gujarati | 2 (<0.1%) |
| Bulgarian | 2 (<0.1%) |
| Estonian | 2 (<0.1%) |
| Arabic (Yemeni) | 2 (<0.1%) |
| Bengali | 2 (<0.1%) |
| Arabic (Egyptian) | 1 (<0.1%) |
| Traditional Chinese | 1 (<0.1%) |
| Fante | 1 (<0.1%) |
| Igbo | 1 (<0.1%) |
| Burmese | 1 (<0.1%) |
| Serbian | 1 (<0.1%) |
| Malayalam | 1 (<0.1%) |
| Amharic | 1 (<0.1%) |
| Swahili | 1 (<0.1%) |
| Turkish | 1 (<0.1%) |
| Arabic (Moroccan) | 1 (<0.1%) |
| East Indian | 1 (<0.1%) |
| Hebrew | 1 (<0.1%) |
| Arabic (Iraqi) | 1 (<0.1%) |
| Arabic (Sudanese) | 1 (<0.1%) |
| Wolof | 1 (<0.1%) |
| Portuguese (Brazilian) | 1 (<0.1%) |

**Appendix 2. Bivariate analyses among those with versus without interpreter use**

| **Demographic** | **No Interpreter**  **(n=2,878) n (%) or Mean ± SD** | **Interpreter**  **(n=2,787)**  **n (%) or Mean ± SD** | **p-value** |
| --- | --- | --- | --- |
| Age (years) | 63.5 ± 18.5 | 59.5 ± 19.0 | <0.001 |
| Sex |  |  | 0.863 |
| Female | 1,255 (50.9%) | 1,209 (49.1%) |  |
| Male | 1,623 (50.7%) | 1,578 (49.3%) |  |
| Race |  |  | <0.001 |
| Asian | 286 (40.6%) | 418 (59.4%) |  |
| Black or African American | 33 (50.8%) | 32 (49.2%) |  |
| Other Race | 1,812 (51.0%) | 1,742 (49.0%) |  |
| White | 705 (56.0%) | 554 (44.0%) |  |
| Not Reported | 42 (50.6%) | 41 (49.4%) |  |
| Ethnicity |  |  | <0.001 |
| Hispanic or Latino | 611 (46.0%) | 716 (54.0%) |  |
| Not Hispanic or Latino | 2,261 (52.3%) | 2,063 (47.7%) |  |
| Not Reported | 6 (42.9%) | 8 (57.1%) |  |
| Preferred Language |  |  | <0.001 |
| Spanish | 2,316 (52.2%) | 2,124 (47.8%) |  |
| Not Spanish | 562 (45.9%) | 663 (54.1%) |  |
| Day of Week |  |  | 0.021 |
| Weekday | 2,085 (49.9%) | 2,094 (50.1%) |  |
| Weekend | 793 (53.4%) | 693 (46.6%) |  |
| Arrival Time |  |  | 0.492 |
| 0:00-05:59 | 222 (51.4%) | 210 (48.6%) |  |
| 06:00-11:59 | 825 (49.3%) | 850 (50.7%) |  |
| 12:00-17:59 | 1,146 (51.7%) | 1,071 (48.3%) |  |
| 18:00-23:59 | 685 (51.1%) | 656 (48.9%) |  |
| ED Disposition |  |  | <0.001 |
| Admitted | 389 (58.1%) | 281 (41.9%) |  |
| Discharged | 1,449 (46.9%) | 1,638 (53.1%) |  |
| Expired | 2 (66.7%) | 1 (33.3%) |  |
| Extended ED Observation | 1,012 (54.5%) | 846 (45.5%) |  |
| Left Against Medical Advice | 18 (54.5%) | 15 (45.5%) |  |
| Transferred | 8 (57.1%) | 6 (42.9%) |  |

*SD, standard deviation; ED, emergency department*

**Appendix 3. Assessment of collinearity**

| **Variable** | **Tolerance** | **VIF** |
| --- | --- | --- |
| Age (years) | 0.917 | 1.090 |
| Sex | 0.990 | 1.010 |
| Arrival time category | 0.997 | 1.003 |
| Weekend arrival | 0.997 | 1.003 |
| Race category | 0.968 | 1.033 |
| Hispanic ethnicity | 0.193 | 5.194 |
| Spanish as preferred language | 0.191 | 5.232 |
| ED Disposition | 0.919 | 1.088 |

*VIF, variance inflation factors; ED, emergency department*

**Appendix 4. Multivariable logistic regression among those with versus without interpreter use**

| **Demographic** | **aOR (95% CI)** | **p-value** |
| --- | --- | --- |
| Age (years) | 0.99 (0.99-0.99) | <0.001 |
| Sex |  |  |
| Female | *reference* | — |
| Male | 1.0 (0.90-1.11) | 0.998 |
| Race |  |  |
| Asian | 1.66 (1.30-2.13) | <0.001 |
| Black or African American | 0.84 (0.50-1.41) | 0.503 |
| Other Race | *reference* | — |
| White | 0.85 (0.74-0.97) | 0.014 |
| Not Reported | 1.09 (0.70-1.69) | 0.715 |
| Preferred Language |  |  |
| Spanish | *reference* | — |
| Not Spanish | 1.01 (0.83-0.1.23) | 0.928 |
| Day of Week |  |  |
| Weekday | *reference* | — |
| Weekend | 0.85 (0.75-0.95) | 0.006 |
| Arrival Time |  |  |
| 00:00-05:59 | 0.98 (0.80-1.21) | 0.863 |
| 06:00-11:59 | 1.09 (0.96-1.24) | 0.208 |
| 12:00-17:59 | *reference* | — |
| 18:00-23:59 | 0.98 (0.86-1.13) | 0.817 |
| ED Disposition |  |  |
| Admitted | 0.68 (0.57-0.81) | <0.001 |
| Discharge | *reference* | — |
| Expired | 0.54 (0.05-6.04) | 0.619 |
| Extended ED Observation | 0.84 (0.75-0.95) | 0.006 |
| Left Against Medical Advice | 0.72 (0.36-1.44) | 0.353 |
| Transferred | 0.55 (0.19-1.57) | 0.263 |

*aOR, adjusted odds ratio; CI, confidence interval; ED, emergency department*
